# A cost-of-illness study of eosinophilic esophagitis in Italy: assessing direct and indirect costs

**DOI:** 10.3389/fgstr.2024.1414251

**Published:** 2024-09-10

**Authors:** Agostino Fortunato, Debora Antonini, Edoardo Vincenzo Savarino, Francesca Racca, Roberto Penagini, Francesca Fanelli, Jean Pierre Saab, Filippo Cipriani, Roberta Giodice, Filippo Rumi, Americo Cicchetti

**Affiliations:** ^1^ Alta Scuola di Economia e Management dei Sistemi Sanitari (ALTEMS) - Università Cattolica del Sacro Cuore, Rome, Italy; ^2^ Department of Surgery, Oncology and Gastroenterology, University of Padua, Padua, Italy; ^3^ Gastroenterology Unit, Azienda Ospedale-Università Padova, Padua, Italy; ^4^ Personalized Medicine, Asthma, and Allergy, IRCCS Humanitas Research Hospital, Milan, Italy; ^5^ Gastroenterolgy and Endoscopy Unit, Fondazione Istituti di Ricovero e Cura a Carattere Scientifico (IRCCS) Cà Grande Ospedale Maggiore Policlinico, Milan, Italy; ^6^ Market Access Department, Sanofi S.p.A, Milan, Italy; ^7^ ESEO Italia - Associazione di famiglie contro l’esofagite eosinofila, Rome, Italy

**Keywords:** eosinophilic esophagitis, unmet medical need, cost-of-illness analysis, social burden, diagnostic delay

## Abstract

**Background:**

Eosinophilic esophagitis (EoE) is a chronic and progressive type 2 inflammatory disease affecting the esophagus. Its prevalence has increased in recent years due to increased awareness, evolving clinical guidelines, and heightened sensitivity among healthcare professionals managing the condition. The exact causes behind EoE’s development remain unknown, and its clinical presentation varies, often leading to significant diagnostic delays depending on the age at which symptoms manifest. Consequently, achieving long-term disease control through heightened awareness becomes imperative. EoE generates a significant clinical burden, resulting in substantial economic consequences for patients, healthcare systems, and society. This study aimed to assess the economic and social impacts on EoE patients within the Italian context.

**Methods:**

A cost-of-illness analysis was conducted from two perspectives: the National Health System (NHS) and the societal perspective. This analysis encompassed direct healthcare, indirect healthcare, and non-healthcare costs. Data were collected and assessed through a survey administered to a panel of expert clinicians and EoE-affected patients.

**Results:**

Managing EoE incurs a significant burden on healthcare systems, amounting to €6,852.28 per patient per year. The primary cost component appears to be direct costs, comprising 60.73% of the total cost per patient for this condition, while indirect costs contribute to 29.68% of the overall management expenses.

**Conclusion:**

This analysis underscores a substantial financial burden on both the healthcare system and patients affected by eosinophilic esophagitis. It emphasizes the imperative need for a continuous and combined effort from clinicians, patients, and families to promptly recognize symptoms and adaptive behavior to mitigate diagnostic delays.

## Introduction

Eosinophilic esophagitis (EoE) is a chronic and potentially progressive type 2 inflammatory disease of the esophagus that has become increasingly prevalent in recent years (1). It is now the most common cause of chronic esophagitis after gastroesophageal reflux disease (GERD) and is also the primary reason for dysphagia and food impaction in children and young adults.

EoE cases were first observed in the late 1970s, and it was officially characterized as a unique clinicopathologic syndrome in the early 1990s (2, 3). Since then, its awareness has grown significantly, and there has been a substantial rise in the number of diagnosed patients worldwide, making EoE a noteworthy scientific and healthcare challenge. Thus, the incidence and prevalence of EoE have significantly increased over time (4). Notably, during the 2000s, the reported incidence rose by 5 times within a span of 4 years and by 27 times over a 10-year period. This upward trend is attributed to several factors. Firstly, changes in diagnostic criteria played a role. The 2007 criteria required more than 15 eosinophils per high-power field (HPF) in esophageal biopsy and the absence of pathologic GERD. The 2017 evidence-based guidelines stated PPI therapy as part of the EoE continuum and introduced the term “PPI responsiveness esophageal eosinophilia” (5). This may have led to an underestimation of EoE cases in previous years by excluding those responsive to PPIs or with GERD. Secondly, early life exposures such as antibiotic use during the first year, admission to neonatal intensive care units, maternal fever, cesarean delivery, and preterm labor have been linked to an increased risk of EoE (6). Thirdly, the increased use of endoscopy in diagnosing gastrointestinal diseases, coupled with EoE diagnosis based on patient symptoms and endoscopic biopsy, has heightened disease awareness, contributing to the observed increase in incidence and prevalence (7). Fourthly, the prevalence of immune-mediated diseases, including atopic and allergic conditions, is on the rise (8). Genetic studies confirm significant genetic sharing between EoE and other immune-mediated diseases, and a history of atopy or food allergy increases the risk of EoE (9).

The causes leading to the development of eosinophilic esophagitis are not yet known. In susceptible individuals, it is speculated that a cellular, non-IgE-mediated immune response (thus different from most common allergies) addressed to food, inhalants, or pathogenic microbes is involved, leading to an accumulation of eosinophils on the wall of the esophagus (10, 11). However, in addition, it is also important to rule out secondary causes such as inflammatory bowel disease and GERD, which are confounding for proper diagnosis. Eosinophils are immune cells common to other type 2 inflammatory diseases such as asthma, allergies, and nasal polyposis, which is why patients with EoE may also have similar allergic forms. In the available scientific literature, the incidence and prevalence of EoE are characterized by extreme heterogeneity across studies.

Differences in epidemiological data were found in several population features, such as geographical location, ethnicity, gender, and age (12). These differences were confirmed by Arias et al. (13), who reported higher prevalence and incidence rates for North America (30.7 and 5.4 per 100,000 inhabitants/year, respectively) than for Europe (16.1 and 1.7 per 100,000 inhabitants/year, respectively). The prevalence rate ratio by gender was also reported in this study. Specifically, the prevalence of EoE among male patients was 53.8 per 100,000 inhabitants, compared to 20.1 per 100,000 in female patients. Other studies reported that EoE was more prevalent in Caucasians than in other ethnic groups (14). The higher prevalence of eosinophilic esophagitis in male patients, along with investigations into family history, twin concordance, and genome-wide association studies, all point toward the existence of a genetic factor in the development of this condition. In addition to these genetic risk factors, mostly unknown environmental factors, especially in early life, are associated with the development of EoE.

The clinical presentation of EoE varies depending on the age at which it becomes apparent. In infants and young children, the symptoms or signs are often non-specific and may include issues like failure to thrive, feeding difficulties, and vomiting (15). In contrast, adolescents and adults tend to experience symptoms related to esophageal fibrosis, probably linked to disease progression due to diagnostic delay and then due to a longer duration of untreated inflammation. Over 80% of adults with EoE exhibit dysphagia, while 50% experience food impactions (16). Remarkably, approximately 50% of individuals who require emergency removal of an esophageal food impaction through endoscopy are found to have eosinophilic esophagitis (17, 18). People with EoE often underestimate the condition and make adjustments to their eating habits by chewing food thoroughly, opting for softer food choices, and drinking frequently during meals. These adaptive behaviors are one of the reasons potentially contributing to a delay in diagnosis. Typically, adults with EoE are diagnosed an average of 7 years after the onset of their symptoms (19).

The gold standard for diagnosing EoE, as well as the evaluation of the disease in the long term, remains the examination of biopsy samples, which should reveal an increased presence of eosinophils within the esophageal epithelium without concurrent eosinophilic infiltration in the stomach or duodenum (20, 21). However, diagnosing EoE can be challenging for several reasons. Firstly, patients with EoE may present several symptoms with variable severity, such as difficulty in swallowing, food impaction, heartburn, chest pain, and nausea, that are non-specific to EoE and similar to those of other gastrointestinal disorders (e.g., gastroesophageal reflux disease and esophageal motility disorders) (12). In addition, there are currently no non-invasive biomarkers for EoE in order to aid diagnosis (22, 23). Another factor contributing to the challenge of correctly identifying EoE is the use of upper gastrointestinal endoscopy without performing esophageal biopsies since up to a quarter of the patients have a normal-appearing esophagus. Endoscopy is indeed the most common tool used to diagnose EoE (24), and it must be coupled with multiple biopsies although the interpretation of their findings may present challenges (25). Finally, the most updated instruments to assess esophageal function and remodeling to the inflammatory conditions like EoE (i.e., functional lumen imaging probe) are not broadly available (26, 27). Therefore, defining the correct diagnosis can often be difficult, leading to a diagnostic delay. Individuals with eosinophilic esophagitis might experience a discrepancy between their symptoms and the histopathological findings, necessitating multiple evaluations of the disease’s activity (28). The presence of eosinophilia is a crucial factor in diagnosing primary EoE, underscoring the importance of excluding secondary causes of esophageal eosinophilia, including inflammatory bowel disease, celiac disease, GERD, esophageal motility disorders, and extraesophageal eosinophilic gastrointestinal disorders (29).

The immediate treatment objectives encompass relieving symptoms, managing inflammation, and restoring normal esophageal function. To achieve these aims, three primary approaches are employed: elimination diet, pharmacological therapy, and esophageal dilation procedures (30–36). First-line treatment could involve dietary changes and pharmacological therapies. Proton pump inhibitors (PPIs) and topical corticosteroids (TCSs) can be included in treatments. For PPIs, some evidence has emerged in the literature reporting clinical efficacy over EoE. In addition, the mode of action of PPIs in blocking gastric acid secretion has been determined, and this antisecretory effect is presumed to underlie their great efficacy in the treatment of GERD. However, PPIs do not prevent the reflux of non-acidic material, and up to 20% of patients with GERD have symptoms refractory to PPIs (37–43). TCS, on the other hand, aims to decrease the number of eosinophils and reduce inflammation in the esophagus. However, despite these conventional therapies (PPI/TCS), many of EoE patients remain with this substantial unmet medical need (44). Whenever feasible, it is advisable to adopt a multidisciplinary approach to therapy. The multidisciplinary approach to type 2 inflammatory diseases represents a pivotal paradigm in modern healthcare (45). Conditions such as EoE underscore the correlation of various medical domains in managing complex patient profiles. This approach involves collaboration among healthcare professionals from diverse specialties, including gastroenterologists, immunoallergologists, and nutritionists, among others. By integrating expertise from different fields, the aim is to comprehensively address the intricate interactions between inflammatory diseases and their coexisting aspects. The multifaceted nature of type 2 inflammatory conditions necessitates a holistic strategy that goes beyond traditional medical boundaries. Through this approach, practitioners can optimize diagnostic precision, enhance treatment efficacy, and improve overall patient outcomes. Moreover, the promotion of awareness and understanding among healthcare professionals about the multidisciplinary nature of type 2 inflammatory diseases is crucial for fostering a collaborative environment and ensuring that patients receive integrated and patient-centric care.

Considering the difficulties in diagnosing this condition and the uncertainty about the epidemiological estimates of EoE, this study aims, through the development of two questionnaires administered to a panel of clinicians and the patient association, to estimate the economic and social burden for EoE patients in the Italian context.

## Methods

### Study design

To evaluate the economic and social impacts of eosinophilic esophagitis in Italy, a cost-of-illness analysis (COI) has been developed in this article (46–49). Two distinct analytical viewpoints were adopted: the perspective of the National Health System (NHS) and the broader social perspective. Under this dual approach, the study considered a comprehensive range of costs, encompassing both direct healthcare expenses and out-of-pocket expenses, as well as indirect costs. Data for this analysis were collected and quantified through two different surveys. Before administering the surveys, the questionnaire’s format underwent validation by a panel of clinical experts with extensive knowledge about the condition. Subsequently, the survey was conducted among individuals afflicted by the specific condition under investigation and an expert panel of clinicians, to have a comprehensive view of the disease.

### Questionnaire

The questionnaire submitted to the panel of clinicians and the patient association was mainly structured into three sections. The perceived epidemiology of the condition, given the extreme variability of evidence in the literature, challenges in diagnosis, and current therapies were investigated. Similar to the clinicians’ questionnaire, the patients’ questionnaire was also structured into different sections. The first section explored the characteristics of the respondent. The second aimed to identify the direct healthcare costs (visits before diagnosis, esophageal dilatation, emergency room admissions, specialist visits, hospitalizations, blood chemistry tests or diagnostic investigations). The third explored the perspective of indirect costs and out-of-pocket expenses, mainly investigating productivity losses resulting from lost workdays due to the condition.

### Cost drivers

In line with the NHS and societal perspective, the research encompassed direct healthcare costs, out-of-pocket expenditures (OOPs), and indirect costs. Direct healthcare expenditures encompassed all the costs directly associated with the treatment of patients (48), such as pharmaceutical interventions, follow-up assessments (e.g., blood tests and radiographic scans), hospital stays, and follow-up appointments. The estimation of direct healthcare costs relied on the utilization of Italian national tariffs and comprehensive data from existing literature regarding costs specific to the Italian healthcare system. To ensure a comprehensive evaluation of drug therapy, we referenced the transparency lists of the Italian Drug Agency (AIFA) (50, 51) and the Summaries of Product Characteristics (SmPC), respectively, to identify an average posology. For the assessment of expenses related to blood tests, outpatient consultations, specialist visits, and adverse events, we referred to the “Nomenclatore tariffario delle prestazioni di assistenza specialistica ambulatoriale” (52). The evaluation of hospitalization costs involved the utilization of the “Tariffario delle prestazioni di assistenza ospedaliera per acuti – Sistema Diagnosis Related Group (DRG)” (53), which enabled us to identify the appropriate tariff for each hospitalization instance documented in the responses. Indirect costs include all expenses incurred by the patient for the treatment of the condition (48). In this study, OOPs, namely, all non-pharmacological treatments (homogenized foods or other specific foods, galenic preparations, etc.), are paid directly by the patient for the management and monitoring of the condition under investigation. The average cost incurred by patients for the purchase of non-pharmacological products and for getting to the treatment center (in case it is far from the patient’s home) or paying for an overnight stay in a private facility was estimated through the analysis of the data provided within the survey. In addition to OOP expenses, we included the indirect costs, all those expenses incurred from the cessation or reduction of work productivity as a result of the morbidity and mortality associated with the disease (49) and the average cost incurred by the patient to reach the specialized center to receive medical treatment/consultation due to the condition.

Indirect costs were defined according to the “human capital approach.” In the human capital approach, the purpose is to estimate the productivity losses resulting from workdays lost due to the condition of working patients. Therefore, the questionnaire investigated the number of days of work lost due to EoE, with the aim of estimating the social burden of the condition being studied. Moreover, the time loss of the patient due to the condition is strictly related to their potential earnings in the future (54). Therefore, to assess the annual productivity loss by patients, 40 weekly working hours for 52 weeks was assumed, resulting in a total of 2,080 working hours. Then, for the valorization of indirect costs, in accordance with the available literature, four wage categories with different salaries were assumed. In more detail, the four categories identified are head manager, middle manager, office workers, and freelancers (55). Finally, according to the provided answers about the ownership of Law 104/92, the appropriate adjustments to lost workdays were done in the analysis. The Italian Law 104/92, often referred to as “Law 104/92,” provides a number of days of leave for family members of severely disabled dependents. These leaves are known as “permits for the assistance of the disabled” and are provided to ensure the necessary care and assistance for family members with disabilities. In this context, it is worth noting that only patient productivity losses are considered in the following analysis because there was no availability from the questionnaire results of the most comprehensive and complete data on caregiver productivity losses. In the past, EoE was classified as a rare disease, which allowed for the costs associated with its management to be covered by the NHS. However, this rare disease status has recently been revoked, due to its growing in prevalence. This change could have significant consequences for EoE patients, as the costs previously covered by the NHS may now be directly shifted to the patients. Specifically, if EoE no longer qualifies for exemption as a chronic disease, it is likely that the costs associated with therapies and medical treatments will become a direct financial burden for patients. This implies that they may have to personally bear these expenses, contributing financially through OOP payments. This shift could significantly impact the quality of life for patients and their access to necessary treatments, putting a strain on individual financial resources. Moreover, the transition from costs supported by the NHS to those borne by the patient could also influence the choice of treatments and care, as patients might be compelled to make decisions based on financial considerations rather than strictly medical ones. In summary, the revocation of the rare disease exemption for EoE could have significant impacts on the management of the condition and the quality of life for affected patients. Thus, EoE does not fall under either chronic diseases with ticket exemption or rare diseases. In the latter case, the exemption is limited to eosinophilic gastroenteritis (RI0030), where eosinophils may affect various parts of the intestine (stomach, small intestine, colon), excluding the esophagus.

## Results

### Population

Given the extreme variability regarding epidemiological data in the literature, a panel of experienced clinicians was asked to indicate the prevalence of EoE, according to their perception. From the comparison, a patient funnel could be estimated in detail. A prevalence of 1:1,000 was estimated, which means 53,000 patients when weighted on the Italian population older than 12 years (53,200,000) (56). Only 41% of these patients are estimated to have been diagnosed as EoE (21.730). Once diagnosed, 97% of patients receive treatment to manage their condition and 66% are found to be treated with conventional medical therapies (14.342). In these two surveys administered to a panel of experienced clinicians and patient associations, there were 52 and 70 respondents, respectively. In the patient survey, 69 questionnaires were usable for analysis since one respondent did not provide authorization for data processing (Legislative Decree 196/2003, as well as the rules of the European Union GDPR Regulation - 25 May 2018). Clinicians responding to the survey were found to be heterogeneous in referring specialty and were distributed among 7 immunoallergologists (13%), 37 gastroenterologists (71%), 4 internists (8%), and 4 pediatricians (8%). The final sample of the patient responders is composed of 14 (20%) women, 22 (32%) men, and 33 (48%) boys and girls under 18 years of age. The average age of the sample is 21 years (**Table 1**).

**Table 1:** Patient characteristics.

Input	n
Total responders (***n***)	69
Men (***n***, %)	22 (32%)
Women (***n***, %)	14 (20%)
Boys up to 14 years (***n***, %)	6 (9%)
Girls up to 14 years (***n***, %)	27 (39%)
Average age (years)	21
Min (years)	3
Max (years)	54
Median (years)	19
Standard deviation (SD)	11
Average age of men (years)	27
Average age of women (years)	33

### Data input

The clinicians’ questionnaire began by investigating the treatment patterns being used at the beginning of the clinical path and in what proportion patients had been unresponsive, declared not eligible, or showed side effects to treatments. It has been reported that 33.8% of patients started therapy for the treatment of EoE with PPIs, 40% with TCs, and 26.2% with PPIs and TCS in combination. There were 43.6% patients unresponsive to PPI therapy and 5.7% have shown side effects due to therapy, while 15.8% of the respondents were non-responders to TCS and 11.2% were not eligible, leading to uncontrolled EoE. Furthermore, it has been asked how long therapy usually lasts, and it was reported to last 20 months and 24.1 months, respectively, for PPI and TCS. The questionnaire continued investigating in case of non-response to TCS and how the patient with EoE was managed. In 31% of the cases, a diet or a combination of diet and drug therapy or between drug therapies was prescribed. In 14% of the cases, systemic steroids and endoscopy were applied, and in only 10% of the cases, PPIs were administered. Finally, the questionnaire explored, according to the respondents’ clinical experience, how many patients were undergoing at least one esophageal dilatation and how many, following that procedure, had a recurrence of esophageal narrowing. The responses showed that 15.6% of patients had undergone interventional endoscopy and 46% had a relapse.

The patients’ questionnaire investigated more specifically the visits patients made before they were diagnosed with EoE, how many visits they had undergone in the 3 months before the survey, and the number of emergency room access and hospitalizations. It also investigated the number of examinations needed to manage the disease. It was found that nine visits on average are required before the patient can receive a correct diagnosis of the eosinophilic condition and that, in 33% of the cases, it is diagnosed following an emergency room access. In the 3 months preceding the survey, 73.9% of the respondents said they made one to three visits, 10.1% made four to seven visits, and 14.5% said they made none. Emergency room visits were found to be only in 18.8% of the respondents’ cases, while 24.6% of the respondents had at least one hospitalization, with an average of 2. Referring to the examinations, the frequency of their performance was investigated, and it emerged that 62.3% of the responders had undergone at least one examination. The most frequently performed examination was esophagogastroscopy with biopsy in 45% of cases, followed by blood tests in 25% of cases and autoimmunity antibody tests in 10%. The manometry test, fibrolaryngoscopy with swallowing test, and allergy tests have been performed with the same frequency, i.e., 10%.

Thereafter, the survey, to investigate the use of drugs or other EoE-specific products not reimbursed by the NHS initially, asked whether the respondents made use of such products, and in 71.1% of cases, the answer was positive. After that, the respondents were asked to answer how much they spent monthly on the acquisition of such products. In addition, again concerning OOP expenditures, the average distance covered in kilometers to get to the center where the respondent was undergoing treatment, the method of travel, and whether an overnight stay was necessary for the patient (one-way travel was calculated) were asked. The results showed that the preferred method of travel to the center was by car in 75% of patients, by train in 15%, and by plane in only 10%. The average distance was estimated to be approximately 177 km, and in 79.7% of cases, the respondents spent at least 1 night of hotel stay.

Finally, in order to explore indirect costs, the questionnaire investigated the type of profession the respondents were engaged in in order to consider the time loss of the patient due to the condition, even if the caregiver was a parent. There were 34.8% respondents who indicated that they were employees, while 8.7% said they were self-employed. Responders in these classes of workers then stated that they lost 4.6 days per month on average to the management of EoE and that only 11.6% of them were beneficiaries of Law 104. Of these, 47.8% were entitled to an exemption code, of which 43.5% referred to the code “RI0030.”

### Cost-of-illness analysis

#### Direct healthcare costs

Coherent with the direct healthcare cost definition, the surveys identified and measured the following cost inputs: pharmacological treatments, examination, visits, emergency room access, and hospitalization. **Table 2** lists the detailed pharmacological treatments to which patients were subjected and its cost per year. More specifically, the cost per patient was estimated through the cost per year divided by the number of patients (%). In more detail, to estimate the correct cost per patient of drug treatments with PPIs and TCS, an average annual cost was used firstly considering the dosage of each of the main drugs belonging to this class. Afterward, since the cost per milligram was estimated, through the posology, it was possible to determine the annual cost per patient.

**Table 2:** Pharmacological treatment.

Treatment	Patients (%)	Cost per patient (€)
**PPI monotherapy**	33.8	164.4
**TCS monotherapy**	40	1,514.4
**PPI and TCS in combination**	26.2	1,678.8
**Non-responders (TCS)—endoscopy**	15.8	14.10

**Table 3** shows the blood chemistry and radiographic examinations. For each cost driver identified, the number of patients, the number of examinations performed per year, the total annual cost per patient, and the total annual cost were reported. To estimate the right number of blood and radiographic examinations undertaken by patients in 1 year, in the survey, it was asked to indicate the number of tests performed in the last 3 months. Consequently, to define the number of examinations performed in a year, the value of each test category carried out in the last quarter was multiplied by the four quarters in a year.

**Table 3:** Examinations.

Examination	Patients (%)	Cost per patient (€)
**Endoscopic dilatation**	15.6	24.3
**Endoscopic dilatation relapse**	46	11.2
**Blood analysis**	25.00	33.40
**Gastroscopy with biopsy**	45.00	14.10
**Antibody test for autoimmunity**	10.00	71.2
**Manometry**	5.00	67.1
**Fibrolaryngoscopy with swallowing test**	5.00	27.1
**Gastroscopy, eosinophil count**	5.00	27.1
**Allergy test**	5.00	71.2

The same procedure was used to consider the cost per patient per year with reference to hospitalizations and emergency room accesses, i.e., the unit cost of the driver was weighted with respect to frequency, as shown in **Table 4**. Regarding GP visits, on the other hand, an average was conducted with respect to the number of visits to which the respondents underwent, so as to have as accurate data on utilization as possible. For all drivers in the table, so that the results referred to the 3 months prior to the survey, the results were then calculated over an entire year.

**Table 4:** Visits, ED admissions, and hospitalizations.

Admissions	Patients (%)	Cost per patient (€)
**GP visits**	25	75.1
**ED admissions**	18.8	42.9
**Hospitalizations**	24.6	76.9

From the direct cost analysis, the intake and management of patients with EoE resulted in a cost of €4,161.14.

#### Out-of-pocket costs

To estimate OOP costs, the survey explored the following cost inputs: use of drugs or other EoE-specific products not reimbursed by the NHS and transportation costs. To assess the initial cost inputs, we investigated the monthly expenses that patients, on average, incurred for the purchase of treatments and/or other products for the management of their condition, and it emerged that the expenditure was €70 per patient (**Table 5**). At this point, this value was weighted against the rate of respondents who reported incurring out-of-pocket expenses and calculated over the course of 1 year. Subsequently, we also examined the distance that patients travel to reach the treatment center, how they traveled, and whether, due to the distance, it was necessary to stay overnight near the center. This made it possible to estimate transportation costs based on the cost per kilometer for the most used travel modalities and to indicate an average daily lodging cost per patient.

**Table 5:** OOP costs.

OOP costs	Patients (%)		Cost per patient (€)
**Drugs or other EoE-specific therapies not reimbursed by the NHS**	71.1		597.2

Transportation costs	Patients (%)	Cost per km (€)	Cost per patient (€)
**Car**	75	0.25	14.7
**Train**	15	0.23	
**Airplane**	10	0.28	
**Overnight expenses**	20		45.4

The measurement of OOP costs resulted in expenses to be borne by the patient for the management of his or her disease amounting to €657.37 per year.

#### Indirect costs

Consequently, the social burden of EoE was estimated through the human capital method. To assess indirect costs, reflecting the annual wages identified in the literature, patients’ employment status data obtained from the survey were fitted to the four categories of workers previously reported (30). Therefore, when considering only the individuals actively employed as identified in the survey (30 patients), the survey allowed us to calculate the average number of workdays lost per month for each patient, which amounted to 4.6 days. Consequently, the average number of work hours lost per month for each patient was 36.8 h. For individuals benefiting from Law 104/92, we did include a different work hour loss productivity in our analysis.

By combining the data gathered through the survey, we first defined productivity loss for employed patients who were not beneficiaries of Law 104/92 and then extended this analysis to the entire patient sample. **Table 6** provides a summary of the social and economic information used to estimate productivity loss for individuals with EoE.

**Table 6:** Patient productivity loss.

Patient productivity loss (no 104)	% of patients in each job class	No. of patients in each job class	Total hours lost in a month	Productivity loss based on average hourly earnings (€)	Annual productivity loss (€)
Executives	1	0.3	10	411	4,517
Middle management	3.9	1.0	38	1,021	11,233
Employees	38.8	10.3	37.7	5,487	60,357
Freelancers	56.3	14.9	549.3	5,282	58,098
**Total loss**				**12,200**	**134,205**
**Total loss per patient**					**5,060**
Executives	1	0.0	0.5	19	206
Middle management	3.9	0.1	1.7	47	512
Employees	38.8	1.3	17.3	250	2,752
Freelancers	56.3	2.0	25.0	241	2,649
**Total loss**				**556**	**6,119**
**Total loss per patient**					**1,760**

From the analysis, an annual productivity loss for working patients with EoE equal to €6,119 and €134,205, respectively, emerged with and without Law 104/02. By weighting the results of the total loss per patient of workers with Law 104 and without by the respective percentage distributions of respondents and then weighing the results on respondents who are actually employed, it is possible to estimate the cost of productivity losses, which is €2,033.8 per year.

#### Summary findings

**Figure 1:**
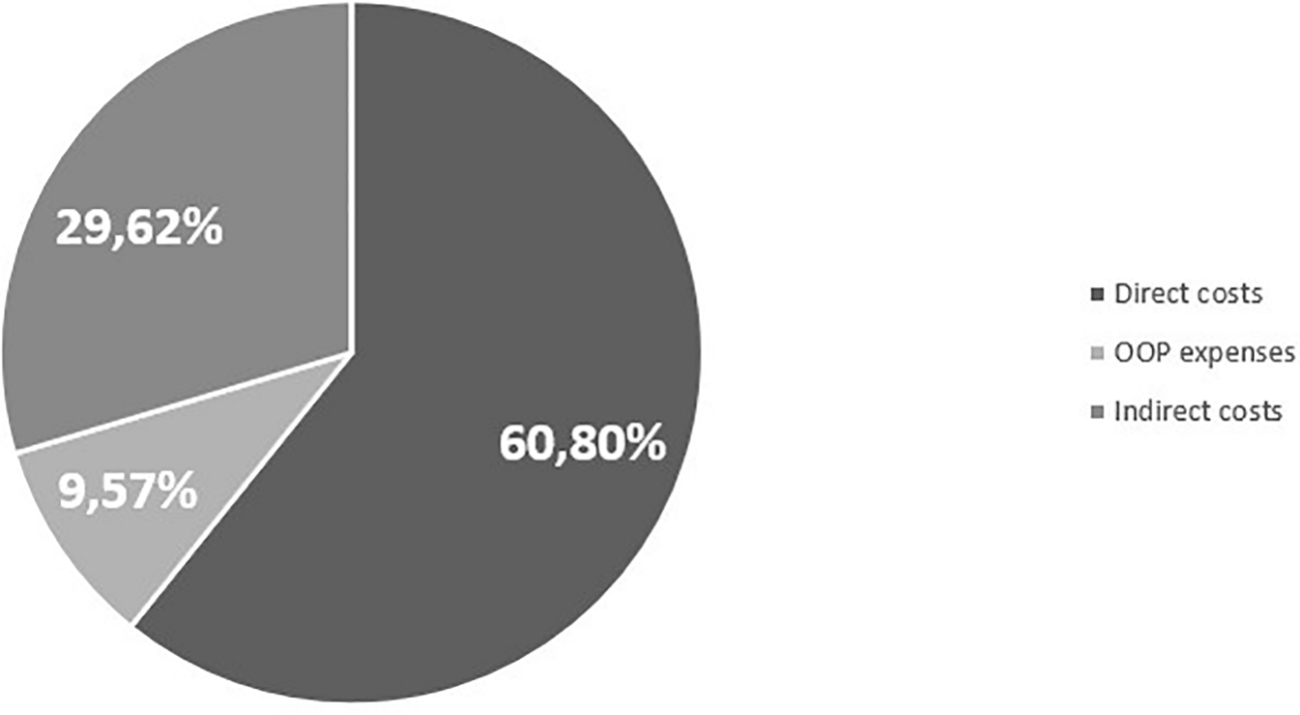
Summary of the cost-of-illness.

In order to fully describe the economic and social impacts of the EoE in Italy, **Table 7** shows the aggregated results per patient of the cost-of-illness analysis, while **Figure 1** shows them in a graphical representation. From the analysis, an EoE cost-of-illness per patient equal to €6,852.3 per year emerged.

**Table 7:** Summary table of cost-of-Illness results per patient.

Input	€	%
Direct costs	€4,174.4	60.8%
OOP costs	€657.4	9.6%
Indirect costs	€2,033.8	29.7%
Total	**€6,856.5**	

## Discussion

The study’s primary objective was to assess the economic and societal implications of EoE in Italy. This research was prompted by the need to address the burden for this disease considering the widespread perception of this unmet medical need. Diagnostic delay is a major problem for EoE, with an average time from the first symptom to the final diagnosis of 7 years. Patient-dependent factors and misdiagnosis are common causes of delay leading to increased EoE severity (7). Through the administration of a survey, it has been revealed that there exists a notable deficiency in immediate responsiveness concerning the condition. This is evidenced by the average requirement of nine visits before reaching the correct diagnosis, as well as the discovery that 33% of patients are diagnosed with EoE solely upon admission to the emergency room. Moreover, the questionnaire allows us to identify and quantify the key direct and indirect cost components, enabling us to subsequently assess the clinical, economic, and societal impacts of the disease within the Italian context. In particular, the study revealed that the total expenditure for managing it amounted to €6,852.3. Notably, the indirect costs associated with drug treatments (€2,033.8) emerged as a significant component of the overall expenditure linked to EoE (29.7%). The direct cost, encompassing GP visits, examinations, and treatment costs, constituted the first-largest component in the assessment, contributing to an overall expenditure impact of 60.7%, equivalent to €4,161.1. Finally, the third major contributor to overall costs was out-of-pocket spending on non-pharmaceutical products, which accounted for 9.6% of the total expenditure. In this context, given the lack of evidence for this area, we found it worthwhile to cite in this article a comparison with another chronic condition with similar management such as GERD. Rossetti et al. (57) found that the average cost per patient-month for GERD was €75.42, with hospitalizations accounting for 34% of the total medical costs. This comparison highlights that while EoE has a significant economic burden, GERD also represents a considerable cost, particularly in terms of hospitalizations and productivity losses, underscoring the importance of comprehensive cost assessments for chronic diseases.

Furthermore, it is important to note that the survey revealed this unmet medical need and that both tertiary centers and clinicians will need to optimize the patient pathway, to minimize the diagnostic delay and identify efficiently EoE patients at an early stage. Thus, improved awareness of the disease could lead to an optimization of the processes and consequently promote early recognition of the patient by decreasing the severity, related costs, and social impact of the disease. This could be reflected in reduced emergency room admissions and fewer hospitalizations, meaning a reduction in workload for hospitals in both organizational terms, reducing waiting lists for example, and economic efficiencies.

The present article, however, is not without limitations. The first concerns the computation of the prevalence rate of EoE in Italy, derived from the clinicians’ perception. In fact, given the paucity of context-specific epidemiological data from Italy on this condition, it was agreed to find evidence in the following methodology. The second, however, lies in the non-inclusion of the caregiver in the analysis of indirect costs, specifically lost productivity, given the lack of specific data on the working status of the caregivers. The third indirect costs related to productivity loss for caregivers especially for pediatric patients have not been considered in the analysis. The fourth consists in adopting a static analysis method, in which costs remain unchanged during different years of study.

## Conclusions

This article highlights a significant financial burden on the healthcare system and on patients affected by EoE. In fact, indirect costs and out-of-pocket expenditure, such as those related to the purchase of drugs, non-pharmacological treatment, and lost working days, represent the most important items of the EoE economic and social burden. The present study could be considered a good starting point for the identification and measurement of the elements affecting the clinical and economic decisions related to the condition. Hence, it can be considered a useful decision support tool for decision-makers, given the limited evidence in the literature regarding the assessment of these aspects, especially in the Italian context, and the considerable heterogeneity of the population investigated. In conclusion, it has emerged that an increase in awareness of EoE has a pivotal role in order to familiarize healthcare professionals with the clinical manifestations of the disease and to streamline early detection with a multidisciplinary approach. This should lead to improvement in the quality of life of patients with EoE and to positive repercussions on the organization of the NHS.

## Data Availability

The original contributions presented in the study are included in the article/**Supplementary Material**. Further inquiries can be directed to the corresponding author.

## Supplementary material

Supplementary Table 1 Clinicians’ survey.

Supplementary Table 2 Patients’ survey.
