# Supplementary material for: A cost-of-illness study of eosinophilic esophagitis in Italy: assessing direct and indirect costs
Source: Front Gastroenterol (Lausanne). 2024 Sep 10;3:1414251. doi: 10.3389/fgstr.2024.1414251 (PMC12952465; doi:10.3389/fgstr.2024.1414251)
Supplement: Supplementary Table 1 — Clinicians’ survey. [file Table1.pdf]

Supplementary Material

Please note that not all questions asked in the surveys were used in the design and development of this analysis, due to their qualitative nature.

Table S1 – Clinicians' survey

1	What is your specialty?
2	Given the extreme variability in prevalence rates and the total absence of Italian data, what is in your perception, the prevalence rate of EoE in Italy, considering both diagnosed and undiagnosed patients?
3	In your clinical experience, given 100 prevalent patients, how many are currently diagnosed?
4	Do you foresee an increase in diagnostic capacity in the near future?
5	If it is yes, for which reasons?
6	What in your clinical experience are the most important "factors - red flags" to enable a correct and timely diagnosis of the patient with eosinophilic esophagitis?
7	How important do you think it is to perform biopsy during endoscopy in under-reported cases
8	In your clinical experience, are there any other factors/red flags to consider for a correct and timely diagnosis of EoE?
9	In your clinical experience, what factors to date interfere with a correct and timely diagnosis of EoE?
10	In your clinical experience, can you assume that all patients diagnosed with EoE undergo drug treatment?
11	If not, given 100 patients you diagnosed, how many are actually treated?
12	In your clinical experience, what proportion of patients are started on therapy with the following drugs? (PPIs as monotherapy)
13	In your clinical experience, what proportion of patients are started on therapy with the following drugs? (TCS in monotherapy)
14	In your clinical experience, what proportion of patients are started on therapy with the following drugs? (PPI and TCS in combination)
15	In light of the reimbursability (TCS) of the only therapy to date indicated for EoE, will this breakdown change in a year's time? If so, how? (PPIs in monotherapy).
16	In light of the reimbursability of the only therapy to date indicated for EoE, will this breakdown change in a year's time? If so, how?(TCS in monotherapy)
17	In light of the reimbursability of the only therapy to date indicated for EoE, will this division change in a year's time? If so, how? (PPI and TCS in combination)
18	In your clinical experience, given 100 patients treated with PPIs first-line, how many do not respond to therapy (%) due to lack of efficacy?

19	How many % patients discontinue PPI treatment because of side effects?
20	In case of a patient responding to PPI therapy, how long does a favorable response to therapy expressed in months last on average?
21	In your clinical experience, what guides you in choosing first-line treatment with PPIs, not specifically indicated for EoE, over treatment with TCS?
22	In your clinical experience, given 100 patients treated first-line with TCS, how many do not respond to therapy (%) due to lack of efficacy?
23	In your clinical experience, given 100 patients with EoE, how many of them are not candidates for TCS?
24	In case of a patient responding to TCS therapy, how long on average does a favorable response to therapy expressed in months last?
25	Do you currently believe that first-line PPI or TCS treatment is equivalent in terms of efficacy?
26	In light of the recent reimbursability (TCS) of the first drug indicated for EoE, do you think PPI treatment will become clinically inappropriate?
27	In case of non-response to TCS, how is the patient with eosinophilic esophagitis managed?
28	In your clinical experience, given 100 patients with EoE, how many undergo at least one esophageal dilatation?
29	Following esophageal dilatation surgery, as a percentage, how many patients experience recurrence of esophageal narrowing?
30	Do you feel that current therapies produce a significant and lasting impact on patients' quality of life and clinical outcomes?
31	On a scale of 1 to 7, how important do you consider each of the following factors to be in assessing treatment efficacy: clinical remission
32	On a scale of 1 to 7, how important do you consider each of the following factors to be in evaluating treatment efficacy: histological remission
33	On a scale of 1 to 7, how important do you consider each of the following factors to be in evaluating treatment efficacy: endoscopic remission

Table S2 – Patients' survey

1	I am: (patient or o caregiver)
2	Indicate please patient's age
3	Indicate please patient's sex
4	Indicate please patient's occupation
5	Which kind of profession?

6	In which Regione do you live?
7	How many work/school/university days (even thinking about online classes or smart working) have you missed on average in the last 3 months for EoE?
8	Please indicate whether the lost days, indicated in the previous question, refer to:
9	Are you a beneficiary of Law 104/92?
10	Are you a beneficiary of an exemption code? If yes, which one?
11	Do you are autonomous in the management of EoE:
12	If yes, does your caregiver benefit from Law 104/92?
13	Indicate the employment status of the caregiver:
14	Profession of the person assisting you (answer only if you answered “Employee” or “Freelancer” in the previous question)
15	In the past three months, have caregivers given up days of work to assist you because of eosinophilic esophagitis?
16	If yes, how many work days have they given up in the past three months?
17	What are the main costs that you and your client have incurred in dealing with this condition?
18	Do you or your caregiver use specific medications or other products because of your condition that are not reimbursed by the National Health Service?
19	If yes, please indicate an average monthly expenditure to purchase products specifically for Eosinophilic Esophagitis (€)
20	Do you or your caregiver have a specific diet?
21	If yes, have you ever had any problems eating your meal at work or in your free time?
22	Which of the following disorders occur due to EoE?
23	Which of the following disorders occur due to EoE? (Please rank the following disorders according to how they impact the Patient's life)
24	In addition to eosinophilic esophagitis have you or your client been diagnosed with one or more different diseases?
25	If yes, please indicate which of these
26	Have any of your family members or your caregiver ever suffered or suffer from similar conditions?
27	How many times on average during the year do you or your caregiver make a specialist visit because of EoE?
28	Please indicate how many visits you or your caregiver have made in the past three months.
29	During the past year, have you or your caregiver had to resort to emergency room access due to illness?

30	If yes, please indicate the number of accesses
31	If yes, please indicate the reason for accessing the emergency room
32	During the past year have you or your caregiver experienced hospitalizations due to illness?
33	If yes, please indicate the number of hospitalizations in the past year
34	If yes, please indicate the reason for hospitalizations in the past year
35	Negli ultimi tre mesi lei o il suo cliente avete effettuato esami diagnostici o di valutazione (esami di laboratorio, endoscopia, biopsia esofagea, ecc.)?
36	If yes, please indicate the number of laboratory tests performed in the past 3 months
37	If yes, please indicate the type of examination performed in the last 3 months
38	Please indicate the average distance covered in km to travel to the center (consider one way) where you or your caregiver is being treated
39	Please rank according to your usage preferences the following ways to reach the center where you or your caregiver is currently being treated for eosinophilic esophagitis?
40	Do you usually stay overnight in private facilities (hotel, B&B, boarding house, private residence, etc.) because of the visit in the said center?
41	If yes, please indicate the average duration in days of the period spent at the accommodation facility
42	If yes, please indicate the estimated cost during the period spent at the accommodation facility (hotel, B&B, guesthouse, private residence, etc.)
43	Please indicate on a scale of 1 to 7 what you think are the most important unmet health needs associated with the condition eosinophilic esophagitis
44	Please indicate on a scale of 1 to 7 what you think are the main disorders that are associated with your condition
45	Have you or your caregiver experienced any other complaints please indicate which ones
46	Losing confidence in the treatment to which one is subjected
47	Regarding your or your caregiver's condition, do you perceive it as a condition that you live with on a daily basis, or is it intermittent?
48	How would you define the level of severity of the condition?
49	What was the first symptom that you or your client manifested?
50	How long after the first symptom did you or your client obtain a diagnosis?
51	If more than one year indicate the time frame
52	How many visits did you or your client make before arriving at a diagnosis of eosinophilic esophagitis?
53	With which professionals?
54	What were the main difficulties in diagnosing the condition?
55	Once diagnosed, what were the main difficulties in managing the condition?
56	How much does the frequency and mode of acquisition of therapies impact your or your caregiver's quality of life?
57	Did the diagnosis of EoE occur as a result of the patient accessing the emergency room for extreme swallowing difficulty?
58	In your perception, on a scale of 1 to 7 how easy was it to get a diagnosis of Eosinophilic Esophagitis, where 1 is 'extremely easy' and 7 'extremely difficult':
59	In your perception, how do you rate your Quality of Life because of the condition?
60	In your experience, what is the factor that most impacts the quality of life of the EoE Patient?
61	In your experience, what are the most strenuous aspects for a caregiver, a person who has to take care of a son/daughter with this condition?
